# Investigation of CD4 and CD8 T cell-mediated protection against influenza A virus in a cohort study

**DOI:** 10.1186/s12916-022-02429-7

**Published:** 2022-07-21

**Authors:** Tim K. Tsang, Kwok-Tai Lam, Yinping Liu, Vicky J. Fang, Xiaofeng Mu, Nancy H. L. Leung, J. S. Malik Peiris, Gabriel M. Leung, Benjamin J. Cowling, Wenwei Tu

**Affiliations:** 1 WHO Collaborating Centre for Infectious Disease Epidemiology and Control, School of Public Health, Li Ka Shing Faculty of Medicine, The University of Hong Kong, 21 Sassoon Road, Pokfulam, Hong Kong, Special Administrative Region China; 2 Laboratory of Data Discovery for Health, Hong Kong Science and Technology Park, New Territories, Hong Kong, Special Administrative Region China; 3 Department of Paediatrics & Adolescent Medicine, Li Ka Shing Faculty of Medicine, The University of Hong Kong, 21 Sassoon Road, Pokfulam, Hong Kong, Special Administrative Region China; 4 HKU-Pasteur Research Pole, School of Public Health, Li Ka Shing Faculty of Medicine, The University of Hong Kong, Pokfulam, Hong Kong, Special Administrative Region China; 5 Centre for Immunology and Infection, Hong Kong Science and Technology Park, New Territories, Hong Kong, Special Administrative Region China

**Keywords:** Influenza, Susceptibility, T cell-mediated immunity

## Abstract

**Background:**

The protective effect of T cell-mediated immunity against influenza virus infections in natural settings remains unclear, especially in seasonal epidemics.

**Methods:**

To explore the potential of such protection, we analyzed the blood samples collected longitudinally in a community-based study and covered the first wave of pandemic H1N1 (pH1N1), two subsequent pH1N1 epidemics, and three seasonal H3N2 influenza A epidemics (H3N2) for which we measured pre-existing influenza virus-specific CD4 and CD8 T cell responses by intracellular IFN-γ staining assay for 965 whole blood samples.

**Results:**

Based on logistic regression, we found that higher pre-existing influenza virus-specific CD4 and CD8 T cell responses were associated with lower infection odds for corresponding subtypes. Every fold increase in H3N2-specific CD4 and CD8 T cells was associated with 28% (95% CI 8%, 44%) and 26% (95% CI 8%, 41%) lower H3N2 infection odds, respectively. Every fold increase in pre-existing seasonal H1N1 influenza A virus (sH1N1)-specific CD4 and CD8 T cells was associated with 28% (95% CI 11%, 41%) and 22% (95% CI 8%, 33%) lower pH1N1 infection odds, respectively. We observed the same associations for individuals with pre-epidemic hemagglutination inhibition (HAI) titers < 40. There was no correlation between pre-existing influenza virus-specific CD4 and CD8 T cell response and HAI titer.

**Conclusions:**

We demonstrated homosubtypic and cross-strain protection against influenza infections was associated with T cell response, especially CD4 T cell response. These protections were independent of the protection associated with HAI titer. Therefore, T cell response could be an assessment of individual and population immunity for future epidemics and pandemics, in addition to using HAI titer.

**Supplementary Information:**

The online version contains supplementary material available at 10.1186/s12916-022-02429-7.

## Background

Influenza virus infections cause considerable morbidity and mortality each year [1], while occasional influenza pandemics can cause a greater impact [2]. Vaccination is currently the most effective strategy for controlling influenza epidemics, but vaccine effectiveness has been suboptimal in some years [3]. Current inactivated influenza vaccines act primarily by inducing humoral immune responses against the head of the hemagglutinin protein of vaccine strains [4]. On the other hand, T cells mediate cellular immune responses and can target proteins that are more conserved across strains [5] and are a target for some universal influenza vaccines [6].

The role of T cell-mediated immunity in providing cross-protective immunity to influenza has been studied extensively in animal models [7–10], but those results may not be applicable to humans. In the last two decades, the role of CD8 T cell-mediated immunity against influenza virus infections in humans has been studied extensively [11], but the role of CD4 T cells is less well understood. The first evidence in humans was from challenge studies, suggesting that pre-existing T cells could be protective against symptom severity or viral shedding [12, 13], but results from experimental exposures to infection may not be generalizable to natural exposures. Previous studies suggested that CD8 response was protective against severe avian influenza A (H7N9) virus infection [14]. Two community-based cohort studies suggested that pre-existing CD8 response was protective against viral shedding and symptom severity after infection in the pandemic influenza A (H1N1) virus outbreak in 2009 [15, 16]. However, the protective effect of T cell-mediated immunity against seasonal influenza virus infections is still unclear, as it is expected that individuals may have a certain degree of humoral immunity.

We conducted a community-based cohort study of families with children from 2009 to 2013 and collected pre- and post-epidemic sera and pre-epidemic peripheral blood mononuclear cells (PBMCs) for the first wave of pandemic influenza and five subsequent influenza A epidemics from 2009 to 2013. Our aim was to determine the association between pre-epidemic levels of CD4 and CD8 T cell response and protection against seasonal influenza A virus infection, and whether these protections were independent of the protection associated with HAI titer. We used inactivated viruses to recall T cell responses rather than using peptide-specific epitopes to provide an overall estimate of the virus-specific T cell responses.

## Methods

### Study design

We conducted a community-based randomized controlled trial (ClinicalTrials.gov NCT00792051) to evaluate the direct and indirect benefits of influenza vaccination [17, 18]. In the pilot study [17], before the 2008/2009 influenza season, we enrolled 119 households each of which had at least one child 6–15 years of age. In each household, one child within this age range was randomized to receive either a single dose of trivalent inactivated influenza vaccination (TIV) or saline placebo. Serum specimens from red-topped tubes were collected at the start of the study, and after 6 and 12 months, from all participants, and in addition, a post-vaccination serum specimen was collected after 1 month from the children who received a vaccine or placebo. Heparinized whole blood samples were also collected at 6 months after the start of the study.

In the main study [18] in 2009/2010, between August 2009 and February 2010, we enrolled 796 households with at least one child 6–17 years of age, including 83 households that had also participated in the pilot study. One child 6–17 years of age in each household was randomized to receive either a single dose of TIV or saline placebo [18]. Serum specimens were collected from all participants at the start of the study and after 12 months, but only in 25% of the participants after 6 months, and in addition, a post-vaccination serum specimen was collected after 1 month from all the children who received the vaccine or placebo. The direct vaccine effects for the pilot and the main studies were reported in previous analyses [17, 18]. Heparinized whole blood samples were also collected at 6 months after the start of the study.

In the subsequent observational follow-up of the same cohort participants from late 2010 to late 2013 without intervention, serum specimens were collected from all participants each autumn (October to December), and 25% of participants also provided serum and whole blood specimens each spring (April to May). The serum specimens collected at the start of the pilot (2008 autumn) were ignored since the first available measurement of CD4 and CD8 T cell response was at the 2009 spring. The post-vaccination serum specimens in the trials were ignored since they were only available to the subset of randomized children. Receipt of influenza vaccine outside of the trial was recorded annually.

### Measurement of humoral immunity

Serum specimens from the same participants were tested in parallel by hemagglutination inhibition (HAI) assays in serial doubling dilutions from an initial dilution of 1:10 using standard methods [18, 19]. The reciprocal of the highest dilution of serum that completely prevents hemagglutination was regarded as the antibody titer. Serum specimens were tested against the novel pandemic influenza A/California/7/2009 (“pH1N1”) and seasonal influenza A/Perth/16/2009-like (“H3N2”) strains. In years 3 and 4 (2011/2012 and 2012/2013), serum specimens were tested against A/California/7/2009 (pH1N1) and A/Victoria/361/2011-like (H3N2). They were the vaccine strains in that year, which were supposed to be dominant.

### Measurement of cellular immunity

Influenza virus-specific CD4 and CD8 T cells in PBMCs isolated from heparinized whole blood were measured by intracellular cytokine staining assay as established before [20–23]. Briefly, PBMCs were incubated with heat-inactivated influenza virus seasonal A/Brisbane/59/2007(“sH1N1”), A/California/7/2009 (pH1N1), A/Perth/16/2009 (H3N2), and A/Victoria/361/2011-like (H3N2) separately (Table 1) at a multiplicity of infection (MOI) of 2 in RPMI medium without serum. The negative control was PBS, and the positive control was *Staphylococcus* enterotoxin B (SEB, 10 μg/ml, Sigma-Aldrich, St. Louis, MO). After 1 h of virus adsorption, cells were resuspended in RPMI medium supplemented with 10% fetal bovine serum. Co-stimulation was done with anti-CD28 and anti-CD49d mAbs (3 μg/ml; BD Biosciences, San Jose, CA, USA). After 12 h of incubation, brefeldin A (Sigma, St. Louis, MO, USA) was added at a final concentration of 10 μg/ml, and PBMCs were incubated for an additional 6 h. Cells were washed with PBS and stained with a mAb mixture consisting of anti-CD3-FITC, anti-CD8-APC, and anti-CD69-Pacific Blue (BD Biosciences, San Jose, CA, USA) for surface markers. Then, cells were fixed and permeabilized by FACS lysing and permeabilazation solutions (BD Biosciences) and stained with IFN-γ-PE intracellularly. Isotype-matched control mAbs of irrelevant specificity were included in all experiments. 5 × 10^5^ events (cells) were collected and analyzed by FACS (BD Biosciences) for each sample. FACS data were prepared for statistical analysis using the FlowJo software (Tree Star, San Carlos, CA, USA). After lymphocyte gating, the CD3^+^CD8^−^ and CD3^+^CD8^+^ cells were gated and referred to as CD4 and CD8 T cells, respectively. CD69^+^IFN-γ^+^ cells within CD4 and CD8 were considered as influenza virus-specific CD4 and CD8 T cells (Additional file 1: Fig. S1).

**Table 1:** The six epidemics and their pre-existing CD4 and CD8 T cell responses in our study

Influenza pandemic/epidemics	The CD4/CD8 T cell response				Identification of infection in sera	
	Time for measurement	sH1N1 strain	pH1N1 strain	H3N2 strain	Rounds for measurement	Influenza strain
1: 2009 autumn, pH1N1 (pandemic)	2009 spring (R1)	A/Brisbane/59/2007	NA	A/Perth/16/2009	R1+R2+R3	A/California/7/2009
2: 2010 autumn, H3N2 (epidemic)	2010 spring (R3)	A/Brisbane/59/2007	A/California/7/2009	A/Perth/16/2009	R3+R4+R5	A/Perth/16/2009
3: 2011 spring, pH1N1 (epidemic)	2010 spring (R3)	A/Brisbane/59/2007	A/California/7/2009	A/Perth/16/2009	R4+R5+R6	A/California/7/2009
4: 2012 spring, H3N2 (epidemic)	2011 spring (R5)	A/Brisbane/59/2007	A/California/7/2009	A/Perth/16/2009	R6+R7+R8	A/Victoria/361/2011
5: 2013 spring, pH1N1 (epidemic)	2012 spring (R7)	A/Brisbane/59/2007	A/California/7/2009	A/Victoria/361/2011-	R8+R9+R10	A/California/7/2009
6: 2013 autumn, H3N2 (epidemic)	2012 spring (R7)	A/Brisbane/59/2007	A/California/7/2009	A/Victoria/361/2011	R8+R9+R10	A/Victoria/361/2011

The CD4 and CD8 T cell responses against sH1N1, H3N2, and pH1N1 for each year were measured by testing the collected PBMCs on an annual basis against the recommended vaccine strains in that year.

### Statistical analysis

To explore associations between pre-existing influenza subtype-specific CD4 and CD8 T cell responses with infection risk of influenza virus, we first identified relevant influenza epidemics from 2009 to 2013 based on local influenza surveillance data, since the influenza epidemics period would be irregular in the tropical and subtropical regions such as Hong Kong [24]. We computed a proxy of influenza activity in the community by using the weekly proportion of outpatient consultations with influenza-like illnesses multiplied by the weekly subtype-specific laboratory detection rates in the Public Health Laboratory Services Branch of the Centre for the Health Protection [25], to identify influenza epidemics during the study period.

Following the previous approach [26–28], we used a 4-fold or greater rise in HAI titers to indicate serological evidence of infections. For each individual and each epidemic, the most recent serum specimen collected prior to that epidemic was used to obtain the pre-epidemic HAI titers, and the earliest serum after that epidemic was used to obtain the post-epidemic HAI titers. Individuals who were vaccinated as part of the trials, or self-reported receipt of vaccination in any year, were excluded from the analyses of that year, since vaccination can also cause a ≥ 4-fold rise that is indistinguishable from those caused by infections. The most recent measurement of CD4 and CD8 T cell responses prior to the epidemic were used as pre-existing CD4 and CD8 T cell responses.

For each pair of subtype-specific CD4 and CD8 T cell responses and each epidemic, we compared the CD4 and CD8 T cell response for infected and uninfected participants by the Wilcoxon signed-rank test. Then, we used generalized estimating equation logistic regression to estimate the association between the CD4 and CD8 T cell responses and infection risk, to account for the fact that individuals could participate in the study with > 1 epidemic and the household clustering among participants. In the regression, we used epidemic-specific intercepts to reflect the difference in infection risk among epidemic, age group (0–17 vs 18+) and HAI titers were adjusted. Since lower infection odds implied higher protection, we defined protection as 100% × (1 − odds ratio). We defined homosubtypic protection as the subtype of T cell response matched with the subtype for the epidemic, since the strain-specific antibody response was long-lived [29, 30]. We defined cross-strain protection as the pair with T cell response against sH1N1 and pH1N1 epidemic. Other pairs were defined as heterosubtypic protection.

The laboratory tests for CD4 and CD8 T cell responses were separately conducted annually. Hence, there could be some heterogeneity in measurements across years. Therefore, we conducted the abovementioned analysis by using standardized CD4 and CD8 T cell responses, calculated by using the *z*-score of CD4 and CD8 T cell responses for each individual (i.e., number of standard deviations from the mean of T cell response in that epidemic). We also used the same approach to explore if such association may only be valid in individuals with low HAI titers defined as HAI titers < 40. We conducted another sensitivity analysis that assumed the protective effect of HAI titer followed a log-linear model as suggested by previous studies [31, 32]. Statistical analyses were conducted using R version 4.0.5 (R Foundation for Statistical Computing, Vienna, Austria).

## Results

**Fig. 1:**
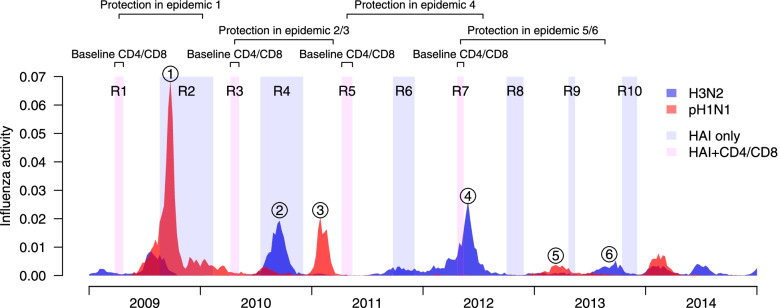
Timelines of our study and local influenza activity for pH1N1 (red) and H3N2 (blue) virus from 2009 to 2013. Shaded regions represented the period for the 10 rounds of serum collections (R1 to R10) and 4 rounds of whole blood collections (R1, R3, R5, and R7). The gray period indicated the rounds with CD4 and CD8 measurements. Arrows indicated the pre-existing CD4 and CD8 response and the corresponding epidemic

There were 10 rounds of serum collection in the study (Additional file 1: Fig. S2), and the CD4 and CD8 T cell responses were measured in the PBMCs collected from 4 periods in the spring of 2009–2012 (Fig. 1). Based on local surveillance data, we identified 6 major epidemics during the study period, including the pH1N1 pandemic outbreak in 2009, two pH1N1 epidemics in 2011 and 2013, and three H3N2 epidemics in 2010, 2012, and 2013 (Fig. 1). The period from which the CD4 and CD8 T cell measurement was used to evaluate the protection, and the sera used to identify infections, for each epidemic, are summarized in Table 1. After excluding those who had received vaccination prior to that epidemic, we analyzed data from 114 participants for the pH1N1 pandemic outbreak and 159–196 participants in the other 5 epidemics. Participants included in the analyses joined both the pilot and the main studies.

The distribution of age and the proportion of HAI titer < 40 among these 5 epidemics were similar (Table 2). The infection risk by age group for these 5 epidemics ranged from 4 to 12%. For the first wave of pH1N1, all individuals had HAI titer < 40, and the infection risk was much higher (18–31%). The participants included in this analysis were similar to the participants in the cohort study for the 5 epidemics (Additional file 1: Table S1).

**Table 2:** The demographic information for the participants in different influenza seasons included in this study

Year	2009	2010	2011	2012	2013	2013
Influenza epidemic type/subtype	pH1N1 (pandemic)	H3N2 (epidemic)	pH1N1 (epidemic)	H3N2 (epidemic)	pH1N1 (epidemic)	H3N2 (epidemic)
Number of individuals	114	135	164	161	196	195
Age group						
< 18 years	49 (43%)	47 (35%)	69 (42%)	68 (42%)	81 (41%)	81 (42%)
18–50 years	58 (51%)	75 (56%)	80 (49%)	76 (47%)	94 (48%)	93 (48%)
≥ 51 years	7 (6%)	13 (10%)	15 (9%)	17 (11%)	21 (11%)	21 (11%)
Pre-season HAI titer < 40 against corresponding epidemic strain	114 (100%)	94 (70%)	118 (72%)	101 (63%)	148 (76%)	128 (66%)
Number of Infections by age groups						
< 18 years	35 (31%)	6 (4%)	10 (6%)	19 (12%)	6 (3%)	6 (3%)
18–50 years	19 (17%)	9 (7%)	7 (4%)	17 (11%)	7 (4%)	4 (2%)
≥ 51 years	1 (1%)	3 (2%)	2 (1%)	3 (2%)	1 (1%)	1 (1%)

### Influenza virus-specific T cell responses

**Fig. 2:**
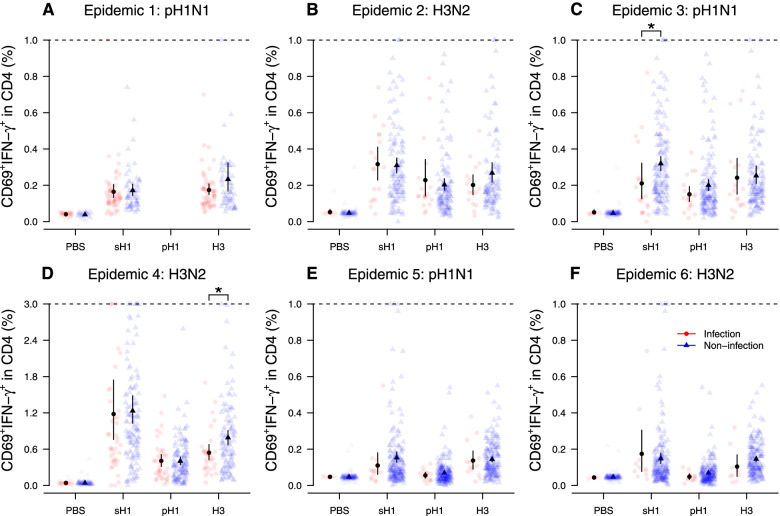
The pre-existing CD4 responses among infected and uninfected individuals in the first pandemic wave of pH1N1 (**A**) and the five epidemics of pH1N1 (**C**, **E**) and H3N2 (**B**, **D**, **F**) in 2010–2013. Star indicates a statistically significant difference with a *p*-value < 0.05 by Wilcoxon the signed-rank test. The T cell response was measured using whole blood samples collected in R1, R3, R3, R5, R7, and R7 for **A**–**F**, respectively

**Fig. 3:**
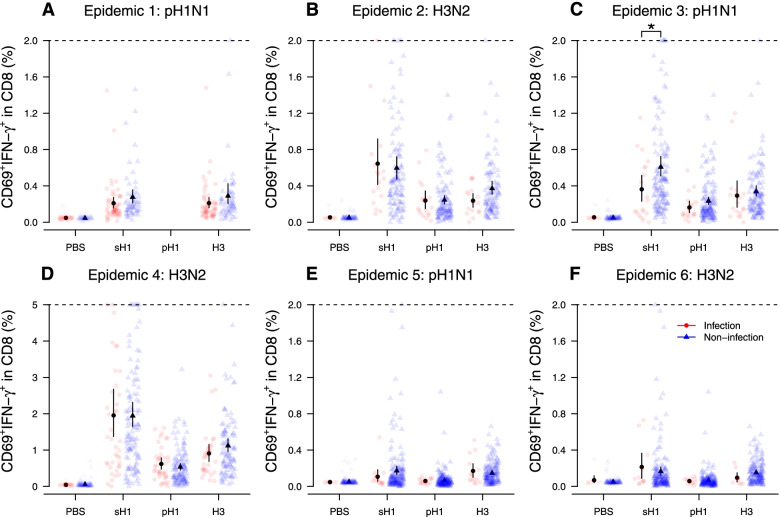
The pre-existing CD8 responses among infected and uninfected individuals in the first pandemic wave of pH1N1 (**A**) and the five epidemics of pH1N1 (**C**, **E**) and H3N2 (**B**, **D**, **F**) in 2010–2013. Star indicates a statistically significant difference with a *p*-value < 0.05 by the Wilcoxon signed-rank test. The T cell response was measured using whole blood samples collected in R1, R3, R3, R5, R7, and R7 for **A**–**F**, respectively

**Fig. 4:**
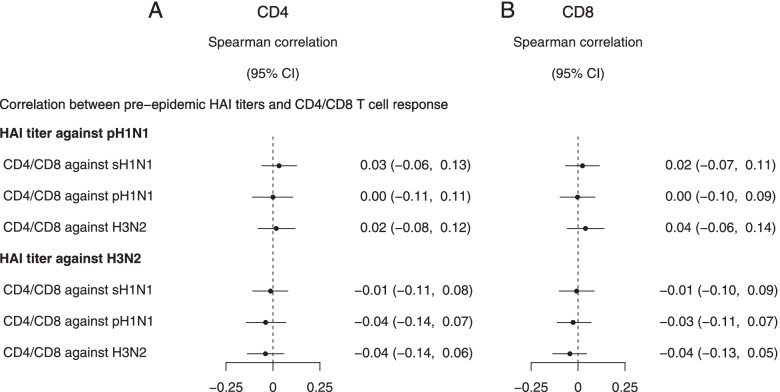
Spearman correlation between pre-epidemic HAI titers and CD4 (**A**) or CD8 (**B**) T cell response

The distribution of influenza virus subtype-specific CD4 and CD8 T cell responses for infected and uninfected individuals were measured by the percentage of CD69^+^IFN-γ^+^ cells within CD4 (Fig. 2) and CD8 (Fig. 3) T cells after PBMCs stimulated with different subtypes of influenza viruses. The distribution of pre-epidemic HAI titer for infected and uninfected individuals is shown in Additional file 1: Fig. S3. For CD4 T cell response, we identified two significant associations: (1) lower pre-existing sH1N1-specific CD4 T cell response in infected participants compared with that in uninfected individuals in the 2011 pH1N1 epidemic (Fig. 2C) and (2) lower pre-existing H3N2-specific CD4 T cell response in infected participants compared with that in uninfected individuals in 2012 H3N2 epidemic (Fig. 2D). For CD8 T cell response, we found a significantly lower pre-existing sH1N1-specific CD8 T cell response in infected participants compared with that in uninfected individuals in the 2011 pH1N1 epidemic (Fig. 3C). There was no correlation between the pre-epidemic HAI titers and CD4 or CD8 T cell response (Fig. 4).

### Homosubtypic protection associated with pre-existing CD4 and CD8 T cell responses

**Fig. 5:**
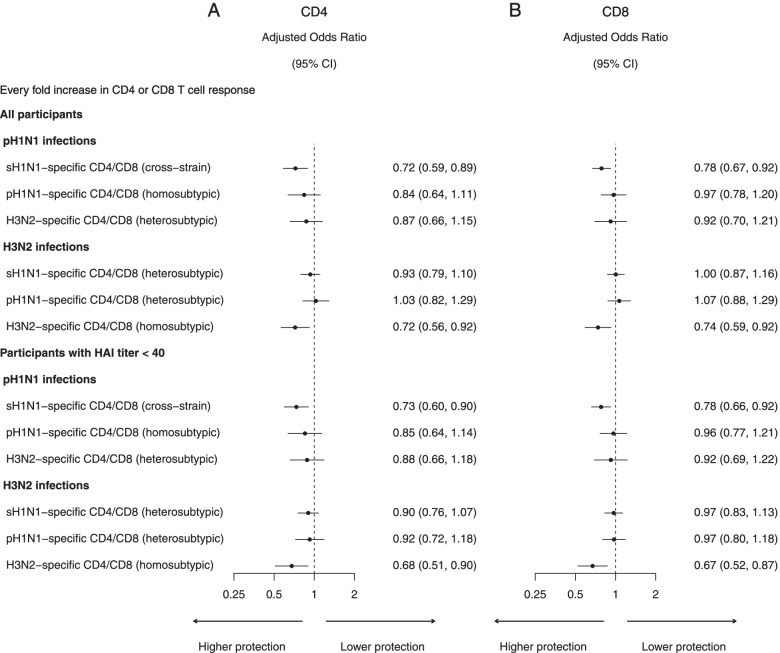
The odds ratios for influenza virus infection for every fold increase in influenza subtype-specific CD4 (**A**) and CD8 (**B**) T cell response for sH1N1, pH1N1, and H3N2 estimated by logistic regression, adjusted for age groups, pre-epidemic HAI titer, and difference in infection risk for epidemics for pH1N1 (2009 pandemic, 2011 and 2013 epidemic) and H3N2 (2010, 2012, 2013 epidemic). Same analyses were also conducted in a subgroup of individuals with pre-epidemic HAI titer < 40

Based on the three H3N2 epidemics in our study period (Fig. 5), the odds ratio of infection for every fold increase in pre-existing H3N2-specific CD4 and CD8 T cell responses was 0.72 (95% CI 0.56, 0.92) and 0.74 (95% CI 0.59, 0.92), corresponding to 28% (95% CI 8%, 44%) and 26% (95% CI 8%, 41%) protection, respectively, adjusted for age, pre-epidemic HAI titer, and the differences in infection risk among epidemics. Among participants with HAI titer < 40, the odds ratio of infection for every fold increase in pre-existing H3N2-specific CD4 and CD8 T cell responses was 0.68 (95% CI 0.51, 0.90) and 0.67 (95% CI 0.52, 0.87), corresponding to 32% (95% CI 10%, 49%) and 33% (95% CI 13%, 48%) protection, respectively, adjusted for age and the differences in infection risk among epidemics. Based on the two pH1N1 epidemics, there were no associations between pre-existing pH1N1-specific CD4 and CD8 T cell responses and infections (Fig. 5). In a sensitivity analysis that assumed the protection associated with HAI titer followed a log-linear model, the degree of homosubtypic protection was similar (Additional file 1: Fig. S4). We used standardized measurement of the T cell responses to conduct these analyses, and the degree of homosubtypic protection was also similar (Additional file 1: Fig. S5).

**Fig. 6:**
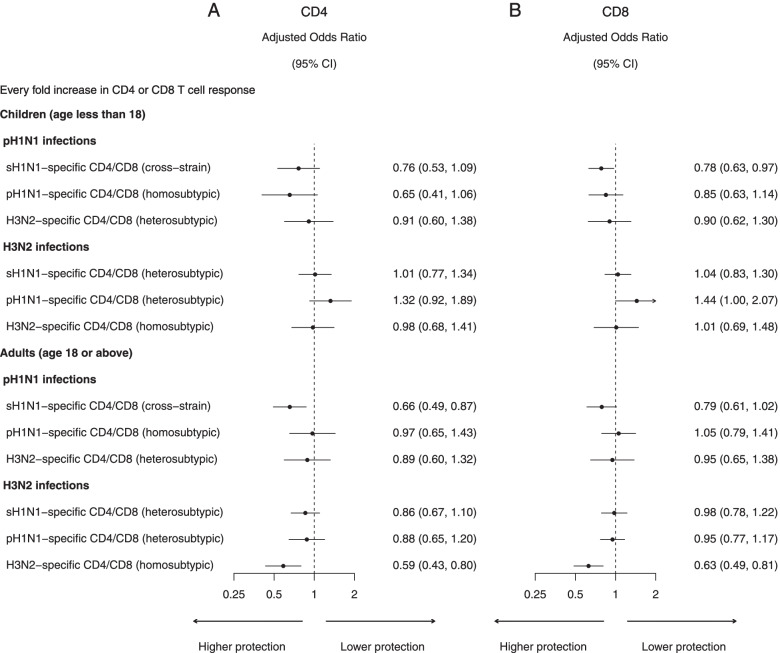
The odds ratios for influenza virus infection for every fold increase in influenza subtype-specific CD4 (**A**) and CD8 (**B**) T cell response for sH1N1, pH1N1, and H3N2 estimated by logistic regression for children (age < 18) and adults (age ≥ 18), adjusted for pre-epidemic HAI titer and difference in infection risk for epidemics for pH1N1 (2009 pandemic, 2011 and 2013 epidemic) and H3N2 (2010, 2012, 2013 epidemic). Arrow in confidence intervals indicates that the upper bound was higher than 2 (which is the limit of the *x*-axis)

We estimated these protections for children and for adults (Fig. 6) and found that the odds ratio of infection for every fold increase in pre-existing H3N2-specific CD4 and CD8 T cell responses was 0.59 (95% CI 0.43, 0.80) and 0.63 (95% CI 0.49, 0.81), corresponding to 41% (95% CI 20%, 57%) and 37% (95% CI 19%, 51%) protection, respectively, for adults, adjusted for pre-epidemic HAI titer and the differences in infection risk among epidemics. However, these protections for children were not significant. Based on the two pH1N1 epidemics, there was no association between pre-existing pH1N1-specific CD4 and CD8 T cell responses and infections in children or adults (Fig. 6). We used standardized measurement of the T cell responses to conduct these age group-specific analyses, and the degree of homosubtypic protection was similar (Additional file 1: Fig. S6).

### Cross-strain and heterosubtypic protection associated with pre-existing CD4 and CD8 T cell responses

For cross-strain protection, based on the pH1N1 pandemic and subsequently two epidemics, we estimated that the odds ratio of pH1N1 infection for every fold increase in pre-existing sH1N1-specific CD4 and CD8 T cell responses was 0.72 (95% CI 0.59, 0.89) and 0.78 (95% CI 0.67, 0.92), corresponding to 28% (95% CI 11%, 41%) and 22% (95% CI 8%, 33%) protection, respectively, adjusted for age, pre-epidemic HAI titer, and the differences in infection risk among epidemics (Fig. 5). Among participants with HAI titer < 40, the odds ratio of infection for every fold increase in pre-existing sH1N1-specific CD4 and CD8 T cell responses was 0.73 (95% CI 0.60, 0.90) and 0.78 (95% CI 0.66, 0.92), corresponding to 27% (95% CI 10%, 40%) and 22% (95% CI 8%, 34%) protection, respectively, adjusted for age and the differences in infection risk among epidemics (Fig. 5). In a sensitivity analysis that assumed the protection associated with HAI titer followed a log-linear model, the result was similar (Additional file 1: Fig. S4). We used standardized measurement of the T cell responses to conduct these analyses, and the results were also similar (Additional file 1: Fig. S5).

We estimated the cross-strain protection for children and for adults (Fig. 6). For adults, based on the pH1N1 pandemic and subsequently two epidemics, we estimated that the odds ratio of pH1N1 infection for every fold increase in pre-existing sH1N1-specific CD4 T cell responses was 0.66 (95% CI 0.49, 0.87), corresponding to 34% (95% CI 13%, 51%) protection, adjusted for pre-epidemic HAI titer and the differences in infection risk among epidemics. For children, based on the pH1N1 pandemic and subsequently two epidemics, we estimated that the odds ratio of pH1N1 infection for every fold increase in pre-existing sH1N1-specific CD8 T cell responses was 0.78 (95% CI 0.63, 0.97), corresponding to 22% (95% CI 3%, 37%) protection, adjusted for pre-epidemic HAI titer and the differences in infection risk among epidemics. Using the same regression model, we also estimated that the odds ratio of H3N2 infection for every fold increase in pre-existing pH1N1-specific CD8 T cell responses was 1.44 (95% CI 1.00, 2.07). We used standardized measurement of the T cell responses to conduct age group-specific analyses, and the results were similar (Additional file 1: Fig. S6).

There was no association between pH1N1 or H3N2 infection and pre-existing CD4 and CD8 T cell responses for the virus of a different subtype, suggesting no heterosubtypic protection (Fig. 5). In a sensitivity analysis that assumed the protection associated with HAI titer followed a log-linear model, we also did not detect heterosubtypic protection (Additional file 1: Fig. S4). We used standardized measurement of the T cell responses to conduct these analyses and also did not detect heterosubtypic protection (Additional file 1: Fig. S5). No heterosubtypic protection was detected in age group-specific analysis using original measurement of the T cell responses (Fig. 6) or using standardized measurement of the T cell responses (Additional file 1: Fig. S6).

## Discussion

Our analyses suggested that there was homosubtypic protection against H3N2 infection associated with pre-existing H3N2-specific CD4 and CD8 T cell responses in three epidemics. We also found evidence of cross-strain protection against pH1N1 infection associated with pre-existing sH1N1-specific CD4 and CD8 T cell responses. This is the first large community cohort for identifying a protective role of T cell immunity against influenza virus infection (including asymptomatic infections) for humans in influenza epidemics. Our results were also consistent with a previous challenge study which suggested that cytotoxic T cell responses > 10% were associated with the absence of infection determined by viral shedding [13]. Animal models suggested that these protections could be independent of other lymphocytes through the production of IFN-gamma [20, 33, 34]. A community-based cohort identified protection against symptomatic influenza A virus infection from T cell response to the H3N2 virus, but this was measuring protection against both infection and illness [15]. Other previous analyses focused on the evidence of protection associated with CD4 and CD8 T cell responses, against illness in humans [12, 13, 16, 35] or from animal models [7–9].

One uniqueness of our study was that we used the inactivated influenza viruses which contain all viral proteins including HA and NA to recall the pre-existing CD4 and CD8 T cells, and therefore, the measured CD4 and CD8 T cell responses would be expected to have an association with protection against infections. Using inactivated viruses to recall T cell responses was better for using the peptide-specific epitopes because we still did not know all the epitopes for each stain of virus and using inactivated whole virus would cover as many as possible epitopes. However, the presence of the internal proteins, such as PB1, PB2, PA, and NS proteins in the inactivated viruses, will be scarce as they are mainly generated as part of the replication process. Thus, the influenza virus-specific T cells presented in the current study may mainly represent the pre-existing CD4 and CD8 T cell responses to surface proteins of the virus, such as HA, NA, and M1 proteins. It is known that antibody responses to vaccination or infection are directed mostly toward epitopes from viral surface-exposed proteins such as HA and NA, whereas epitopes recognized by cellular immunity may be broadly derived from both internal and surface proteins [36]. The major CD4 T cell epitopes were derived from HA protein, which could be processed by the antigen-presenting cell from inactivated virus and presented on MHCII to the CD4 T cells. In contrast to changes that occurred at the HA antibody-binding site, the rest of the HA protein was relatively conserved within the subtypes of influenza A. Therefore, the assay in the current study measured the homosubtypic protection by mainly reflecting the T helper type 1 response to the HA and would provide B cell help for the antibody response to any of the strains within the subtypes [37]. However, there were virtually no CD8 T cell epitopes within HA and NA; hence, the CD8 T cell responses to the virus were going to be largely derived from the internal and matrix proteins [36, 38, 39]. While the naïve CD8 T cell response was dependent on antigen processing of viral proteins derived from live virus and presentation of those epitopes on MHC I, antigen cross-presentation can re-stimulate CD8 T cell immunologic memory from prior exposure to the virus and may explain the similar results using live and inactivated virus. Hence, CD8 T cells cannot protect against infection, and their response may simply be measuring the overall robustness of the immune response to influenza rather than a direct correlate of protection against infection. Therefore, although we found a similar association with infection risk for CD4 and CD8 T cell response, it should be interpreted as the protective effects were mainly from the CD4 T cell response.

On the other hand, previous studies detected the pre-existing T cells specific for individual influenza virus internal and matrix proteins by using overlapping peptides [12, 13, 15, 16]. As the major CD8 T cell epitopes are derived from internal and matrix proteins of influenza viruses [36, 38, 39], the correlates of protection against illness and severity found in those studies would not predict protection against infection. Indeed, two community studies did not detect protection against pH1N1 infections [15, 16] but found evidence of protection against illness after infections. A study on patients hospitalized with pH1N1 infections also found that the CD4 and CD8 T cell responses were lower for those severe cases, compared with those with mild infections [35].

We found pre-existing CD4 and CD8 T cell responses for sH1N1 virus were associated with the protection against antigenically very divergent pH1N1. This was consistent with the epidemiological evidence from the past pandemics that previous seasonal H1 influenza virus infections were associated with a lower infection risk with newly emerged pandemic H2 strains [40, 41]. Also, our previous study demonstrated that the bulk memory cytotoxic T lymphocytes established by sH1N1 infections who have not been exposed to the pH1N1 virus can directly lyse pH1N1 virus-infected target cells in healthy adult volunteers [20], supporting this association.

The identified protection against infections was similar for individuals with an HAI titer < 40 indicating limited humoral immunity against infection [32]. This suggested that pre-existing influenza virus-specific T cells were protective even in the absence of detectable HAI antibodies. The CD4 and CD8 T cells may also be cytotoxic and directly contributed to protection [5, 42, 43]. This suggested there could be heterogeneity in infection risk among individuals with undetectable antibodies, for example, a portion of the elderly was not infected although they had no immunity suggested by HAI titers [44]. Therefore, such T cell-mediated immunity is particularly important in a pandemic setting when most individuals have no immunity against the newly emerged strain, either lowering infection risk or severity [16, 40]. Usually, only HAI antibody is measured as a correlate of protection. Measuring T cell response could be a valuable approach to evaluate population immunity at the beginning of pandemic, in addition to HAI titers [45] to guide policy, as HAI titers was shown to be imperfect correlate of protection [31].

We attempted to determine the protection associated with T cell response for children and for adults. Although this analysis may be underpowered, we found that the homosubtypic protection against H3N2 infection was associated with H3N2-specific T cell response in adults but not for children, which was consistent with a previous analysis suggesting that there could be protection other than HAI titers for adults but not for children [46]. On the other hand, we observed an increased heterosubtypic risk of H3N2 infection of pre-existing pH1N1-specific CD8 T cell response. We interpreted this as a type I error in the analysis since there was no biological reason for this increased risk.

The cross-reactive T cell is one of the strategies for the development of universal influenza vaccines such as a universal T cell vaccine [47], since it is supposed to provide broad protection against many strains [5, 6]. While we did not detect heterosubtypic protection, this did not rule out the heterosubtypic protection against severe disease, which may still have clinical relevance [34]. We found CD4 and CD8 T cell response for pre-pandemic sH1N1 was associated with protection against pH1N1 infection, suggesting that subtype- or HA group-specific vaccine with a wider breadth of protection could be a promising first step toward universal influenza vaccine [6, 48].

Our study has some limitations. First, we did not have the pH1N1-specific CD4 or CD8 T cell measurement prior to the 2009 pH1N1 pandemic, and therefore, the sample size was insufficient to estimate its protection effect. Second, influenza virus infections were identified by using ≥ 4-fold rises in consecutive HAI titers, which may miss infections with 2-fold rises [49]. While using this definition is not optimal, it is a gold standard method to estimate the influenza attack rate in the human population and widely adopted for > 70 years [49]. Third, since it was a serology cohort, therefore no information related to severity, such as duration of episodes of infection, was available. Therefore, we may not evaluate the protection against disease severity associated with T cell response. Furthermore, we did not collect respiratory swabs in the study, but CD8 T cells were found enriched in the lung compared to the blood [50]. Therefore, the CD8 T cell response in our study may not be optimal. Fourth, since it was a cohort study with a long follow-up period, there could be variation in the degree of exposure among the study participants, so that some individuals may not have been exposed to the influenza virus in our study. Therefore, there could be a variation in the association between different epidemics. Hence, we also combined those epidemics in the analysis, by using epidemic-specific intercepts in the logistic regression. Fifth, for each influenza epidemic, we excluded participants with vaccination prior to that epidemic but ignored vaccination history in earlier years. Hence, our analyses assumed that earlier influenza vaccinations had no effect on the association between T cell response and infection risk. Although we did not explicitly include the infection status in the prior epidemic in estimation, we included pre-epidemic HAI titers in the model, which may partly explain the protection from previous infections. Sixth, our study measured CD4 and CD8 T cell responses from April to May each year. Therefore, the pre-existing CD4 and CD8 T cell response may be measured about a half year earlier than the start of some epidemics, although those periods had minimal influenza activity based on surveillance data. Seventh, we did not consider the role of influenza B in this study and assumed influenza A and B were independent. Finally, since our study is observational, we adjusted for age group and influenza epidemic in our analysis, but we cannot rule out other potential confounders in detected associations between infection risk and the pre-existing CD4 and CD8 T cell responses, such as antigenic sin, or imprinting may play a role of these associations, particular for sH1N1.

## Conclusions

We showed homosubtypic protection associated with pre-existing CD4 and CD8 T cell responses, against infections in H3N2 epidemics. Also, we showed evidence that pre-existing CD4 and CD8 T cell responses to sH1N1 were associated with protection against pH1N1 infection. This could be important for the development of a universal influenza vaccine or assessment of population immunity for future epidemics and pandemics.

## Supplementary Information

**Additional file 1: Figure S1**. Gating strategy and representative data of FACS analysis for influenza virus-specific CD4 and CD8 T cells. **Figure S2**. Flowchart of the cohort studies and the collection of serum samples and whole blood samples. **Figure S3**. The HAI titer distribution before and after the first pandemic wave of pH1N1 and the five epidemics of pH1N1 and H3N2 in 2010-2013. **Figure S4**. The odds ratios for influenza virus infection for every fold increase in standardized influenza subtype-specific CD4 and CD8 T cell response for sH1N1, pH1N1 and H3N2 estimated by logistic regression. **Figure S5**. The odds ratios for influenza virus infection for every fold increase in standardized influenza subtype-specific CD4 and CD8 T cell response for sH1N1, pH1N1 and H3N2 estimated by logistic regression. **Figure S6**. The odds ratios for influenza virus infection for every fold increase in standardized influenza subtype-specific CD4 and CD8 T cell response for sH1N1, pH1N1 and H3N2 estimated by logistic regression for children (age <18) and adults (age ≥18). **Table S1**. The demographic information for the participants, including those excluded in the analysis.

## Data Availability

The datasets used and/or analyzed during the current study are available from the corresponding author on reasonable request. Code for analysis is available at GitHub: https:// github.com/timktsang/T_cell_protection.

## Abbreviations

H3N2: Seasonal influenza A (H3N2) virus
PBMCs: Peripheral blood mononuclear cells
pH1N1: Pandemic influenza A (H1N1)pdm09 virus
sH1N1: Seasonal influenza A (H1N1) virus
