# Supplementary material for: Investigation of CD4 and CD8 T cell-mediated protection against influenza A virus in a cohort study
Source: BMC Med. 2022 Jul 21;20:230. doi: 10.1186/s12916-022-02429-7 (PMC9301821; doi:10.1186/s12916-022-02429-7)
Supplement: Supplementary file 1 — Additional file 1: Figure S1. Gating strategy and representative data of FACS analysis for influenza virus-specific CD4 and CD8 T cells. Figure S2. Flowchart of the cohort studies and the collection of serum samples and whole blood samples. Figure S3. The HAI titer distribution before and after the first pandemic wave of pH1N1 and the five epidemics of pH1N1 and H3N2 in 2010-2013. Figure S4. The odds ratios for influenza virus infection for every fold increase in standardized influenza subtype-specific CD4 and CD8 T cell response for sH1N1, pH1N1 and H3N2 estimated by logistic regression. Figure S5. The odds ratios for influenza virus infection for every fold increase in standardized influenza subtype-specific CD4 and CD8 T cell response for sH1N1, pH1N1 and H3N2 estimated by logistic regression. Figure S6. The odds ratios for influenza virus infection for every fold increase in standardized influenza subtype-specific CD4 and CD8 T cell response for sH1N1, pH1N1 and H3N2 estimated by logistic regression for children (age <18) and adults (age ≥18). Table S1. The demographic information for the participants, including those excluded in the analysis. [file 12916_2022_2429_MOESM1_ESM.docx]

**Additional File 1**

**Investigation of CD4 and CD8 T cell-mediated protection against influenza A virus in a cohort study**

Tim K. Tsang^1,2^, Kwok-Tai Lam^3^, Yinping Liu^3^, Vicky J. Fang^1^, Xiaofeng Mu^3^, Nancy H. L. Leung^1,2^, J. S. Malik Peiris^1,4,5^, Gabriel M. Leung^1,2^, Benjamin J. Cowling^†1,2^, Wenwei Tu^†3^

^†^ These authors jointly supervised this work

**Affiliations:**

1. WHO Collaborating Centre for Infectious Disease Epidemiology and Control, School of Public Health, Li Ka Shing Faculty of Medicine, The University of Hong Kong, Hong Kong Special Administrative Region, China.

2. Laboratory of Data Discovery for Health, Hong Kong Science and Technology Park, New Territories, Hong Kong Special Administrative Region, China.

3. Department of Paediatrics & Adolescent Medicine, Li Ka Shing Faculty of Medicine, The University of Hong Kong, Hong Kong Special Administrative Region, China.

4. HKU-Pasteur Research Pole, School of Public Health, Li Ka Shing Faculty of Medicine, The University of Hong Kong, Hong Kong Special Administrative Region, China.

5. Centre for Immunology and Infection, Hong Kong Science and Technology Park, New Territories, Hong Kong Special Administrative Region, China.

**Corresponding author:**

Prof. Benjamin J Cowling, School of Public Health, Li Ka Shing Faculty of Medicine, The University of Hong Kong, 21 Sassoon Road, Pokfulam, Hong Kong.

Tel: +852 3917 6711; Fax: +852 3520 1945; email: [bcowling@hku.hk](mailto:bcowling@hku.hk)

Prof. Wenwei Tu, Department of Pediatrics and Adolescent Medicine, Li Ka Shing Faculty of Medicine, The University of Hong Kong, 21 Sassoon Road, Hong Kong; Phone: +852 3917 9354; Fax: +852 2819 8142; email: wwtu@hku.hk

**SUPPLEMENTARY FIGURE LEGENDS**

**Figure S1.** Gating strategy and representative data of FACS analysis for influenza virus-specific CD4 and CD8 T cells. After lymphocyte gating, the CD3^+^CD8^-^ and CD3^+^CD8^+^ cells were gated and referred to as CD4 and CD8 T cells, respectively. CD69^+^IFN-γ^+^ cells within CD4 and CD8 were considered as influenza virus-specific CD4 and CD8 T cells. The percentages of CD69+IFN-γ+ cells within CD4 and CD8 after PBMCs stimulated with inactivated influenza viruses (sH1N1, pH1N1 and H3N2) were shown. PBMCs treated with PBS and SEB were the negative and positive controls, respectively.

**Figure S2.** Flowchart of the cohort studies and the collection of serum samples and whole blood samples. Start means beginning of the study year (Sep to Nov), and mid means the mid of that study year (April-May). The round numbers are corresponding to figure 1. R0 and R11 was not shown in figure 1, since the information in these two rounds were not used in the analysis.

**Figure S3.** The HAI titer distribution before and after the first pandemic wave of pH1N1 (Panel A) and the five epidemics of pH1N1 (Panel C and E) and H3N2 (Panel B, D and F) in 2010-2013. For pH1N1, A/California/7/2009 was tested against for all six epidemics. For H3N2, A/Perth/16/2009-like was tested against for epidemics 1-4, and A/Victoria/361/2011-like was tested against for epidemics 5-6.

**Figure S4.** The odds ratios for influenza virus infection for every fold increase in standardized influenza subtype-specific CD4 (Panel A) and CD8 (Panel B) T cell response for sH1N1, pH1N1 and H3N2 estimated by logistic regression, adjusted for age groups, pre-epidemic HAI titer and difference in infection risk for epidemics for pH1N1 (2009 pandemic, 2011 and 2013 epidemic) and H3N2 (2010, 2012, 2013 epidemic). In the analysis, the protection of HAI titer was assumed to follow a log-linear model.

**Figure S5.** The odds ratios for influenza virus infection for every fold increase in standardized influenza subtype-specific CD4 (Panel A) and CD8 (Panel B) T cell response for sH1N1, pH1N1 and H3N2 estimated by logistic regression, adjusted for age groups, pre-epidemic HAI titer and difference in infection risk for epidemics for pH1N1 (2009 pandemic, 2011 and 2013 epidemic) and H3N2 (2010, 2012, 2013 epidemic). Same analyses were also conducted in a subgroup of individuals with pre-epidemic HAI titer < 40.

**Figure S6.** The odds ratios for influenza virus infection for every fold increase in standardized influenza subtype-specific CD4 (Panel A) and CD8 (Panel B) T cell response for sH1N1, pH1N1 and H3N2 estimated by logistic regression for children (age <18) and adults (age ≥18), adjusted for pre-epidemic HAI titer and difference in infection risk for epidemics for pH1N1 (2009 pandemic, 2011 and 2013 epidemic) and H3N2 (2010, 2012, 2013 epidemic). Arrow in confidence intervals indicates that the upper bound was higher than 2 (which is the limit of x-axis).

**Table S1**. The demographic information for the participants, including those excluded in the analysis.

| **Year** | **2009** | **2010** | **2011** | **2012** | **2013** | **2013** |
| --- | --- | --- | --- | --- | --- | --- |
| **Influenza epidemic type/subtype** | **pH1N1 (pandemic)** | **H3N2**  **(epidemic)** | **pH1N1 (epidemic)** | **H3N2**  **(epidemic)** | **pH1N1 (epidemic)** | **H3N2 (epidemic)** |
| Number of individuals | 1409 | 1013 | 1676 | 1371 | 1288 | 1280 |
|  |  |  |  |  |  |  |
| Age group |  |  |  |  |  |  |
| <18 years | 376 (27%) | 280 (28%) | 729 (43%) | 582 (42%) | 556 (43%) | 553 (43%) |
| 18-50 years | 905 (64%) | 650 (64%) | 810 (48%) | 652 (48%) | 603 (47%) | 599 (47%) |
| ≥51 years | 128 (9%) | 83 (8%) | 137 (8%) | 137 (10%) | 129 (10%) | 128 (10%) |
|  |  |  |  |  |  |  |
| Pre-season HAI titer <40 against corresponding epidemic strain | 1407 (100%) | 854 (84%) | 1163 (69%) | 912 (67%) | 899 (70%) | 806 (63%) |
|  |  |  |  |  |  |  |
| Number of Infections by age groups |  |  |  |  |  |  |
| <18 years | 144 (10%) | 27 (3%) | 114 (7%) | 161 (12%) | 44 (3%) | 38 (3%) |
| 18-50 years | 96 (7%) | 62 (6%) | 109 (7%) | 137 (10%) | 38 (3%) | 34 (3%) |
| ≥51 years | 10 (1%) | 8 (1%) | 12 (1%) | 36 (3%) | 5 (0%) | 10 (1%) |
